# REALIS: Postgenomic Analysis of *Listeria Monocytogenes*

**DOI:** 10.1002/cfg.137

**Published:** 2002-02

**Authors:** Uwe Kärst, The REALIS Consortium

**Affiliations:** GBF Ges. f. Biotechnol. Forschung Mascheroder Weg 1 Braunschweig D-38124 Germany

## Abstract

*Listeria monocytogenes* is a remarkably successful food-borne pathogen. It is capable a) of surviving and proliferating under conditions that exist within the food chain, such as at low temperatures, high salt and low pH and b) of colonizing animal host tissues after ingestion of contaminated food, causing opportunistic infections mainly, but not exclusively, in immunocompromised hosts. The ultimate goals of REALIS are two fold: Firstly, it aims to completely decipher all genes required for survival in and adaptation of *Listeria monocytogenes* to two very different environments, ie., the infected host and the external environment. Secondly, using genomics and postgenomic tools, REALIS seeks to precisely address fundamental questions regarding evolutionary relationships between pathogenic and non-pathogenic Listeria and to define qualities of particularly successful clonal pathovariants in causing disease. This project will provide both industry and health care managers with rational approaches to curbing food-borne contamination, minimising risks of infection and providing novel pharmacological approaches for halting the fulminant course of infection.


**Comparative and Functional Genomics**
*Comp Funct Genom* 2002; **3**: 32–34.
**DOI:** 10.1002/cfg.137

Conference Review

### REALIS: Postgenomic analysis of *Listeria monocytogenes*

Uwe Kärst* The REALIS Consortium†
*GBF, Ges. f. Biotechnol. Forschung, Mascheroder Weg 1, D-38124 Braunschweig, Germany*

**Correspondence to:*
*GBF, Ges. f. Biotechnol. Forschung, Mascheroder Weg 1, D-38124 Braunschweig, Germany.*
*E-mail: kaerst@gbf.de*

*†A full list of authors appears at the end of this paper.*

Received: 16 November 2001
Accepted: 4 December 2001
Published online:
20 December 2001

### Abstract

***Listeria monocytogenes*** **is a remarkably successful food-borne pathogen. It is capable a) of surviving and proliferating under conditions that exist within the food chain, such as at low temperatures, high salt and low pH and b) of colonizing animal host tissues after ingestion of contaminated food, causing opportunistic infections mainly, but not exclusively, in immunocompromised hosts. The ultimate goals of REALIS are two fold: Firstly, it aims to completely decipher all genes required for survival in and adaptation of** ***Listeria monocytogenes*** **to two very different environments, ie., the infected host and the external environment. Secondly, using genomics and postgenomic tools, REALIS seeks to precisely address fundamental questions regarding evolutionary relationships between pathogenic and non-pathogenic Listeria and to define qualities of particularly successful clonal pathovariants in causing disease. This project will provide both industry and health care managers with rational approaches to curbing food-borne contamination, minimising risks of infection and providing novel pharmacological approaches for halting the fulminant course of infection. Copyright © 2001 John Wiley & Sons, Ltd.**

*Listeria monocytogenes* is a ubiquitous Gram-positive soil bacterium that is responsible for listeriosis, a food-borne infection that affects humans and animals and that is characterized by a variety of severe clinical manifestations, including meningoencephalitis, septicemia and abortion. The exposure and successful colonization of mammalian hosts is facilitated by the bacterium's tolerance to conditions used to preserve food, i.e. high salt and acidic conditions, respectively, together with the ability to grow at low temperature. Due to the particular lifestyle as intracellular parasites, enabling the bacteria to spread from cell to cell, they are able to breach the blood-brain and the placental barrier, leading to meningitis and abortion. These characteristics together with its capability to grow in standard media, its broad host spectrum, the availability of animal models and tissue culture systems, its completely sequenced genome, and established genetic manipulation protocols make *L. monocytogenes* a very interesting model system for studying intracellular pathogens and T-cell mediated immunity.

Based on previous efforts – the sequencing of the complete genomes of the pathogenic *L. monocytogenes* within the 4th European Framework programme and of the closely related non-pathogenic species *L. innocua* [1] – the REALIS project (part of the 5th European Framework programme) aims at a postgenomic functional analysis of the lifestyle of *Listeria monocytogenes* in the environment and in the infected host. The scientific and technological objectives of the project are:

- the study of the evolution of this pathogenic organism by comparative genomics and the definition of particularly successful clonal pathovariants,
- the development of post genomic strategies to provide a complete picture of the adaptive properties of this food-borne pathogen,
- an improved understanding of the molecular mechanisms by which environmental clues are perceived and translated into adaptive responses,
- the development and establishment of an integrated bioinformatics database incorporating and linking all of the available and produced information
- the development of high throughput strategies and tools for transcriptome analysis, the generation of mutants, and the analysis of *in vivo* gene expression.

Copyright © 2001 John Wiley & Sons, Ltd.

The work on these objectives is organized into six closely interacting work packages (WP) focusing on gene expression analyses, ie., i) transcriptomics, ii) proteomics, iii) the analysis of regulatory units, iv) the generation and characterization of mutants, v) comparative genomics, and vi) bioinformatics.

The transcriptomics work package WP1 focuses on the study of the transcription patterns of all genes of *L. monocytogenes* using macro- and micro-array technology and multi criteria analysis to achieve an understanding of gene expression on a global level. It is intended to answer specific questions and identify genes of interest for further functional analysis, especially genes involved in the different stages of the pathological process, in bacterial dissemination, and in survival in the environment. The expression patterns will be analysed – typically by comparing the wild type strain with a deletion mutant and that mutant complemented with the deleted gene – in combination with proteome analyses, mutant analyses, and comparative genomics. Prototypes of high-density membranes and micro-arrays have been tested and have already yielded evidence for new proteins under the control of the central virulence regulator protein in *Listeria*, PrfA. Production of both membranes and slides has started.

Gene expression is also analysed in the proteomics work package WP2 at the protein level using high-resolution two-dimensional gel electrophoresis and rapid protein identification by peptide mass fingerprinting and mass spectrometric microsequencing. Detailed information is sought on when and in which amounts proteins are synthesized and what their stability is, whether there are post-translational modifications, possibly related to signal transduction, and on the targeting and localization within the bacterial cell. The focus of these investigations in combination with the other work packages is on the subproteomes of secretory, cell wall- and membrane-associated proteins as well as on protein complexes and proteins under PrfA control. Standard 2D PAGE is now being used to prepare protein maps for different subproteomes and new and improved techniques such as Blue Native PAGE and 16BAC/SDS-PAGE are being established and developed to overcome methodological limits. Mapping of the secretory and cell wall-associated proteins is progressing well, and several proteins putatively under positive or negative control of PrfA have been identified.

The central regulon analysis work package WP3 deals with the identification and characterization of regulatory networks and their units (regulons, stimulons) that are involved either directly in the damage to the infected host, i.e. virulence factors *sensu strictu*, or in sensing and transducing environmental conditions which are indicative of *Listeria's* natural habitats or the infected host as well as those prevailing during food production and storage. Current work focuses on PrfA-regulated networks, stress responses controlled by sigma factors, two-component systems and transport systems. Experimental approaches for studying *in vitro* and *in vivo* (cell culture) conditions are expression analyses using RT-PCR, transcription analysis (arrays, WP1), and proteomics (WP2) comparing different environmental conditions, e.g. nutrients and temperature, using mutants created and/or selected directly or indirectly by IVET and STM methods.

The work package WP4 concentrating on the analysis of mutants intends a large-scale generation of mutants that will allow the identification of novel genes required for survival and adaptation in multiple environments or infected hosts. Mutants are created by standard methods and attenuated strains selected using techniques such as signature-tagged mutagenesis. Furthermore, the development of novel tools to address and analyse promoter activity and expression of genes identified by functional transcriptomics, proteomics, and the analysis of stress-controlled regulons are also planned. A considerable number of mutants have already been created that are currently being characterized with regard to phenotype, e.g. growth and infectiousness, and gene function.

The objectives of the comparative genomics work package WP5 are the identification of important new structural and regulatory genes that are part of the general physiology of *L. monocytogenes*, required for its survival in extreme conditions, and contribute to its virulence in various animal models. The analyses are based on the known sequences of the genome of *L. monocytogenes* (strain EGDe, serovar 1/2a), on the genome of the non-pathogenic species *L. innocua* (serovar 6a), the two most closely related species of the genus *Listeria*, and on the partial sequence of a *L. monocytogenes* strain of serovar 4b. In addition, the identification of marker genes present in strains more likely to be involved in clinical cases and epidemics, respectively, is intended, and genes of particular interest will be inactivated as a priority in work package 4. Phylogenetic analyses of *Listeria* species are currently

Copyright © 2001 John Wiley & Sons, Ltd.

being carried out to investigate the genetic heterogeneity and the degree of horizontal gene transfer within the genus *Listeria*.

The bioinformatics work package WP6 defined and set up an integrated bioinformatic database that will play a key role in the integration of the project and in the data flow between the partners. The database is built on Lion Bioscience's SRS6 system and will ensure that the collected data from genomics, transcriptomics, and proteomics can be collected, organized, and integrated to extract a maximal amount of useful information. The tools developed will allow complex queries and reap important added value by combining information from different origins, including external public resources.

### The REALIS Consortium

F. Baquero, M. Baumgärtner, C. Buchrieser, D. Cabanes, T. Chakraborty, P. Cossart, O. Diekmann, E. Domann, O. Dussurget, F. Engelbrecht, H. Fsihi, F. Garcia-del Portillo, P. Glaser, W. Goebel, T. Hain, C. Jacquet, B. Jassal, J. Johansson, B. Joseph, J. Kreft, F. Kunst, L. Jänsch, P. Martin, E. Milohanic, J.-C. Perez-Diaz, P. Rice, M. Sanchez-Ruiz, J. Schaumburg, J.-M. Soulié, M. Scortti, M. Trost, J.-A. Vazquez-Boland, H. Voss, J. Wehland.

### References

1. Glaser P, Frangeul L, Buchrieser C, *et al.* 2001. Comparative Genomics of Listeria species. *Science* **294**: 849-852.

Copyright © 2001 John Wiley & Sons, Ltd. 

## References

[PDF_00209] Glaser P., Frangeul L., Buchrieser C., Rusniok C., Amend A., Baquero F., Berche P., Bloecker H., Brandt P., Chakraborty T. (2001). Comparative genomics of Listeria species.. Science.

